# Late-Onset of Spinal Neurodegeneration in Knock-In Mice Expressing a Mutant BiP

**DOI:** 10.1371/journal.pone.0112837

**Published:** 2014-11-18

**Authors:** Hisayo Jin, Naoya Mimura, Makiko Kashio, Haruhiko Koseki, Tomohiko Aoe

**Affiliations:** 1 Department of Anesthesiology, Chiba University Graduate School of Medicine, Chiba City, Chiba, Japan; 2 Department of Medicine and Clinical Oncology, Chiba University Graduate School of Medicine, Chiba City, Chiba, Japan; 3 Laboratory for Developmental Genetics, RIKEN Research Center for Allergy and Immunology, Yokohama, Japan; 4 Department of Anesthesiology, Tokyo Women's Medical University, Yachiyo Medical, Center, Yachiyo, Chiba, Japan; Toho University School of Medicine, Japan

## Abstract

Most human neurodegenerative diseases are sporadic, and appear later in life. While the underlying mechanisms of the progression of those diseases are still unclear, investigations into the familial forms of comparable diseases suggest that endoplasmic reticulum (ER) stress is involved in the pathogenesis. Binding immunoglobulin protein (BiP) is an ER chaperone that is central to ER function. We produced knock-in mice expressing a mutant BiP that lacked the retrieval sequence in order to evaluate the effect of a functional defect in an ER chaperone in multi-cellular organisms. Here we report that heterozygous mutant BiP mice revealed motor disabilities in aging. We found a degeneration of some motoneurons in the spinal cord accompanied by accumulations of ubiquitinated proteins. The defect in retrieval of BiP by the KDEL receptor leads to impaired activities in quality control and autophagy, suggesting that functional defects in the ER chaperones may contribute to the late onset of neurodegenerative diseases.

## Introduction

Proteins destined for the secretory pathway are inserted into the ER cotranslationally and subjected to quality control [1]. ER molecular chaperones and folding enzymes such as BiP, calnexin, and protein disulfide isomerase facilitate the correct folding or degradation of these newly synthesized proteins as well as of misfolded proteins [2]. The accumulation of misfolded proteins in the ER beyond the capacity of quality control causes ER stress and induces the unfolded protein response (UPR) [3]. Further ER stress can cause cellular dysfunction and cell death, resulting in diverse human disorders such as neurodegenerative diseases [4].

Mammalian ER luminal chaperones have a carboxyl terminal Lys-Asp-Glu-Leu (KDEL) amino acid sequence, which is recognized by the KDEL receptor in post-ER compartments [5]. ER chaperones and the KDEL receptor are sorted into the transport vesicles coated with coat protein (COP) I complex and retrieved to the ER [6]. Yeast BiP (Kar2p) is essential for survival, while the deletion of the retrieval sequence (in yeast: His-Asp-Glu-Leu, HDEL) is dispensable because the UPR is activated and the loss of the chaperone in the ER is compensated for [7]. The complete depletion of BiP also has lethal effects on mammalian early embryonic cells [8].

In order to elucidate the physiological processes that are sensitive to the retrieval of BiP during development and adulthood in multi-cellular organisms, we previously produced knock-in mice expressing a mutant BiP in which the retrieval sequence was deleted by homologous recombination. The homozygous mutant BiP mice died within several hours after birth due to respiratory failure with impaired biosynthesis of the pulmonary surfactant, especially surfactant protein C, by alveolar type II cells [9]. The heterozygous mutant BiP mice grew up to be apparently normal adults. However, vulnerability to ER stress may exist in the mutant BiP mice, leading to chronic organ injuries. Indeed, some of them displayed motor disabilities in aging. We found a degeneration of some motoneurons in the spinal cord accompanied by accumulations of ubiquitinated proteins. The accumulation of misfolded proteins is one of the most common features in neurodegenerative diseases. Functional defects in the ER chaperones may contribute to the late onset of neurodegenerative diseases.

## Results

### The mutant BiP mice revealed motor disabilities in aging

**Figure 1:**
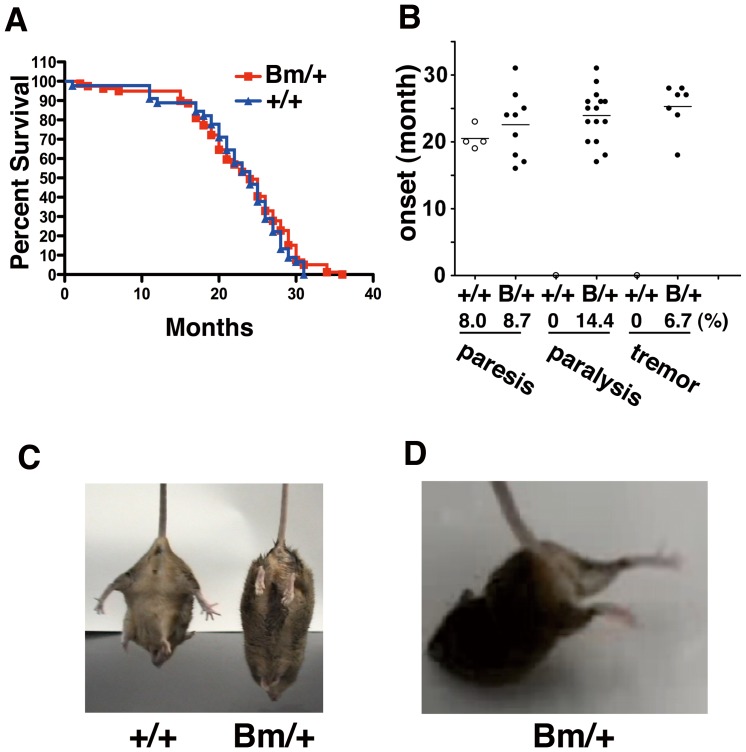
The mutant BiP mice revealed motor disabilities in aging. (A) Kaplan-Meier plots demonstrate that the heterozygous mutant BiP mice (Bm/+, n = 80) live as long as the wild-type mice (+/+, n = 45). (B) Some of the mutant BiP mice displayed paralysis and tremors after they were more than one year old. Heterozygous mutant BiP mice (Bm/+, n = 104) and wild type mice (n = 50) more than 10 months old were observed. No sign of weakness; +/+ 46/50, Bm/+ 73/104, tremor; +/+ 0/50, Bm/+ 9/104, paresis of one hindlimb; +/+ 4/50, Bm/+ 9/104, paralysis of one or both hindlimbs; +/+ 0/50, Bm/+ 15/104. To compare values between two groups, Chi-square and Fisher's exact test was done. Statistical significance was found. P value is 0.0033. (C) A seventeen month-old mutant BiP mouse displayed paralysis and loss of righting reflex. (D) A seventeen month-old mutant BiP mouse displayed paralysis (Video S1).

The heterozygous mutant BiP mice live as long as the wild type mice (Figure 1A). We could not detect a significant motor disability during their young period, using the Rotarod performance test. At very advanced ages, some of the mutant BiP mice displayed paralysis and tremors after they were more than one year old, although a few wild-type mice show partial paresis (Figure 1B). Some of aged mutant BiP mice displayed loss of righting reflex and suffered from paralysis (Figure 1C, D, and Video S1). The ratio of motor disabilities is significantly higher in the heterozygous mutant BiP mice.

### Motoneurons at the anterior horn of spinal cords of aged mutant BiP mice suffer from ER stress

**Figure 2:**
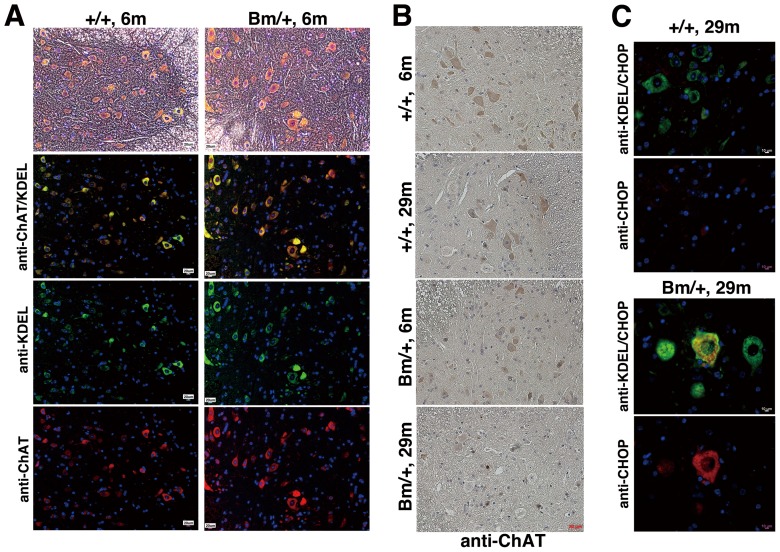
Motoneurons at the anterior horn of spinal cords of aged mutant BiP mice suffer from ER stress. (A) Motoneurons stained by an anti-choline acetyltransferase antibody (red) at the anterior horn in the spinal cord of both a 6 month-old wild type (+/+, 6 m) and a 6 month-old mutant BiP mouse (Bm/+, 6 m) express ER chaperones as well (green). Scale bars, 20 um. (B) The immunoreactivity with an anti-choline acetyltransferase antibody at the anterior horn is reduced in the aged 29 month-old mutant spinal cord (Bm/+, 29 m). Scale bars, 20 um. (C) Large cells at the anterior horn of the aged 29 month-old mutant spinal cord (Bm/+, 29 m) express ER chaperones as well as CHOP. Scale bars, 10 um. The nuclei were stained with Hoechst 33258 (blue, A and C).

**Figure 3:**
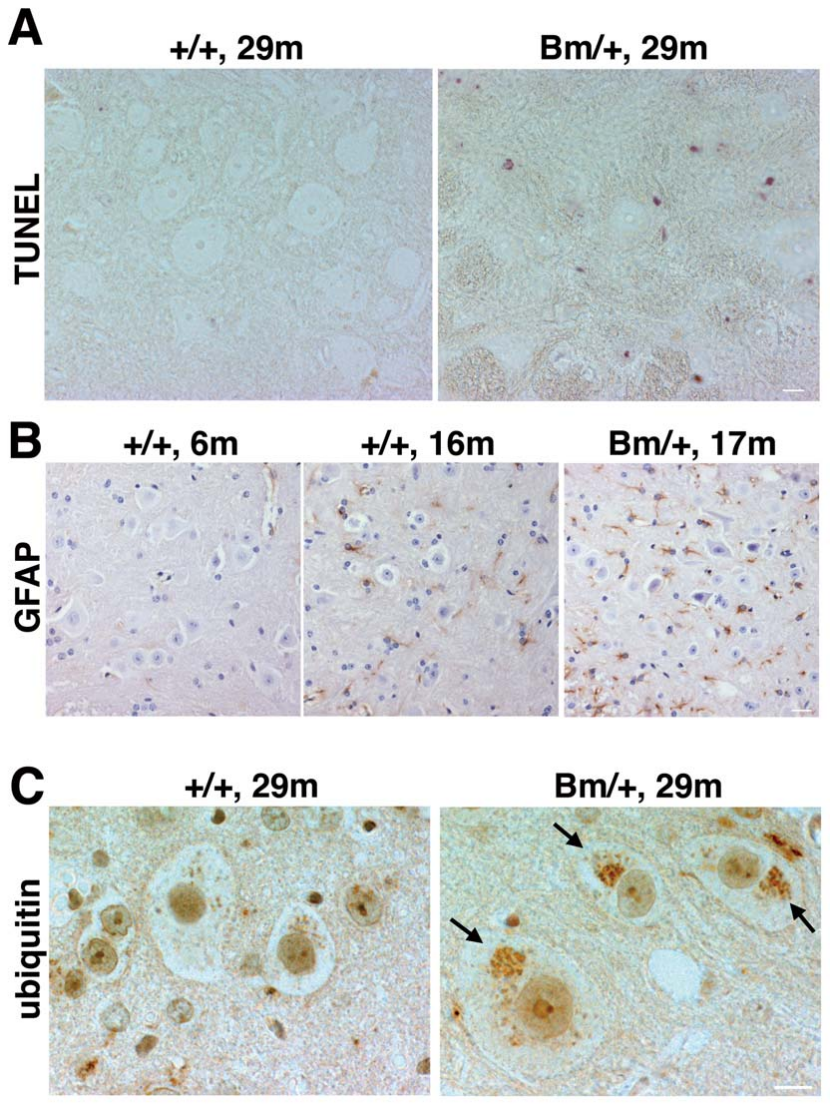
Some motoneurons in the spinal cord revealed a degeneration accompanied by accumulations of ubiquitinated proteins. (A) TUNEL staining revealed some apoptotic cells at the anterior horn in the spinal cord of a 29 month-old mutant BiP mouse (Bm/+, 29 m). Scale bars, 10 um. (B) The immunoreactivity with an anti-GFAP antibody at the anterior horn is increased in a 17 month-old mutant spinal cord (Bm/+, 17 m). Scale bars, 20 um. GFAP positive cells are counted (five fields in each mouse, GFAP positive cells/the number of nucleus). +/+, 16 m; 33/199, 35/174, 34/192, 40/181, 42/207, Bm/+, 17 m; 42/132, 50/140, 59/147, 39/154, 58/143 +/+, 6 m; 5/143, 4/131, 4/141, 0/95, 0/107. The ratio of GFAP positive cells is significantly higher in the mutant BiP spinal cord (Bm/+, 17 m) compared to that in the wild type (+/+, 16 m) by t test (p value is 0.0009). (C) The aggregations were stained by an anti-ubiquitin antibody in the large cells at the anterior horn of the 29 month-old mutant spinal cord (Bm/+, 29 m, arrowheads). Scale bars, 10 um.

In order to investigate whether defective retrieval of the mutant BiP may contribute to the pathogenesis of motor disability, we examined the spinal cords of both the wild type and mutant BiP mice. By staining with an anti-KDEL antibody, we found that some large cells expressed BiP and other KDEL-containing chaperones highly at the anterior horn of the spinal cords of 6 month-old mice of both types. Those cells were also stained with an anti-choline acetyltransferase antibody, suggesting that motoneurons at the anterior horn expressed ER chaperones highly (Figure 2A). Then we examined much older mutant BiP mice with motor disability. Motoneurons at the anterior horn of the spinal cord stained with an anti-choline acetyltransferase antibody in a 29 month-old mutant BiP mouse were less than those in the wild type mice (Figure 2B). We found that some large cells at the anterior horn of the spinal cord of the 29 month-old mutant BiP mouse expressed CHOP, a cell death related transcriptional factor during ER stress. The expression of CHOP was less obvious in the wild type littermate (Figure 2C). In accordance with these findings, terminal deoxynucleotidyl transferase dUTP nick end labeling (TUNEL) staining revealed that some apoptotic cells existed in the mutant spinal cord, accompanied by an enhanced gliosis stained with an anti-glial fibrillary acidic protein (GFAP) antibody (Figure 3A and B). It was difficult to find a TUNEL positive cell in the wild type spinal cord. We counted cells in Figure 3B. The ratio of GFAP positive cells was significantly higher in the mutant BiP spinal cord (Bm/+, 17 m) compared to that in the wild type (+/+, 16 m).

These data suggest that motoneurons at the anterior horn of spinal cords of aged mutant BiP mice suffer from ER stress, resulting in cell death.

### Some motoneurons in the spinal cord revealed a degeneration accompanied by accumulations of protein aggregations

**Figure 4:**
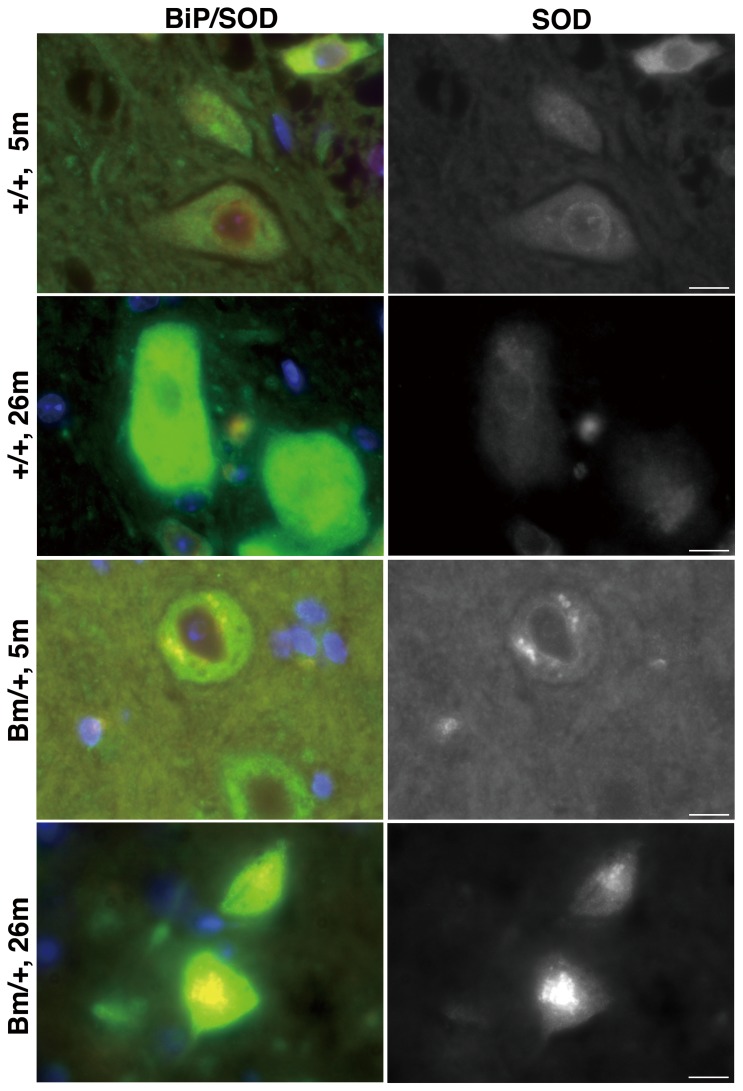
Aggregations were obvious in the aged mutant BiP mouse. The aggregations were evaluated by immunofluorescence microscopy with double labeling by using a rabbit anti-SOD1 antibody (red) and a mouse anti-KDEL mAb for the wild type BiP and other ER chaperones (green, +/+, 5 and 26 month-old), or by using a rabbit anti-SOD1 antibody (red) and a mouse anti-HA mAb (15E6) for the mutant BiP (green, Bm/+, 5 and 26 month-old). Scale bars, 10 um. Aggregations were observed in large cells at the anterior horn of a 26-month-old mutant spinal cord (Bm/+, 26 m).

Protein aggregation and neuronal cell death are hallmarks of various neurodegenerative diseases [10]. We examined protein aggregation in the mutant spinal cord by staining it with an anti-ubiquitin antibody, which revealed cytoplasmic aggregations in the large cells at the anterior horn of the aged mutant BiP mouse (Figure 3C). One possible candidate for the aggregated proteins is superoxide dismutase (SOD) 1 [11]. Point mutations of the human SOD 1 gene are associated with a familial motoneuron disease (amyotrophic lateral sclerosis, ALS) [12]. While we observed fuzzy perinuclear staining with an anti-SOD1 antibody in large cells at the anterior horn of the spinal cords of wild type mice, aggregations were found instead in the cells of the mutant BiP mice. Those aggregations were much more obvious in the aged 26 month-old mutant BiP mouse (Figure 4, Bm/+, 26 m).

**Figure 5:**
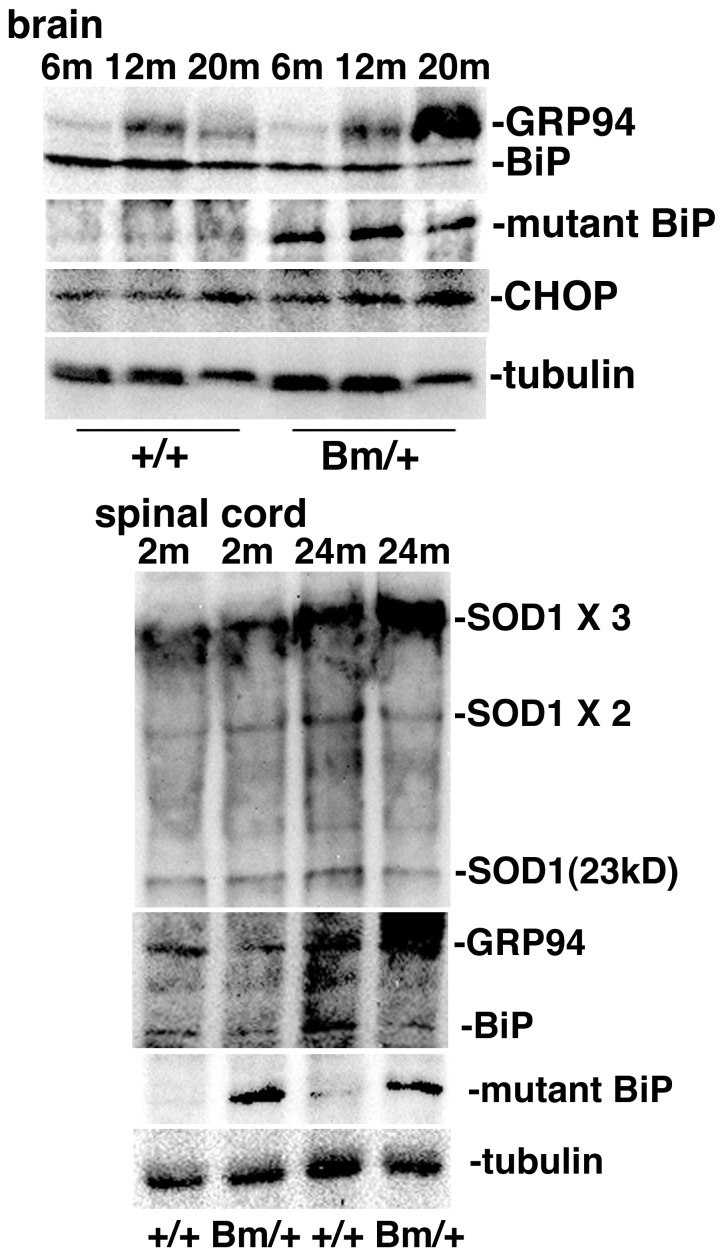
The expressions of chaperones in the mutant BiP mice. The heterozygous mutant BiP mice and the litter mate wild type mice were anesthetized by pentobarbital, and the brains and spinal cords were removed. They were subjected to Western blot analysis with an anti-KDEL mouse mAb for BiP and GRP94, an anti-HA mouse mAb for mutant BiP, an anti-CHOP rabbit antiserum, and an anti-SOD1 rabbit antiserum.

We examined the expression of BiP and the mutant BiP in the brain and spinal cord of the mutant BiP mice by Western blotting in Figure 5. The expression of GRP94, another ER chaperone, seemed to be increased in the aged mutant BiP brain and spinal cord. We also found an accumulation of SOD1 aggregates in the aged mutant BiP spinal cord, which corresponds to the finding in Figure 4.

### Deletion of the retrieval signal from BiP may cause aberrant quality control

**Figure 6:**
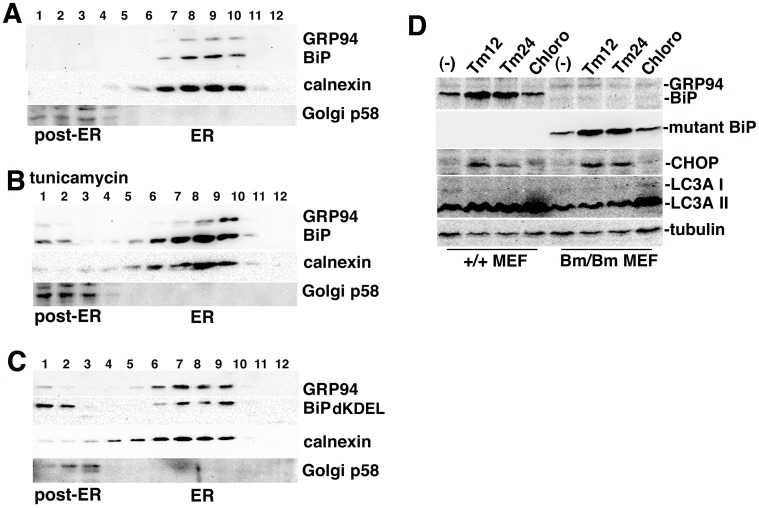
Lack of the KDEL sequence from BiP may lead to some functional defects of the ER chaperone in quality control and autophagy. (A, B) A fraction of BiP is secreted from the ER under stressed conditions. The postnuclear supernatants (PNS) from HeLa cells with (B) or without (A) treatment by tunicamycin 2.5 ug ml^−1^ for 24 h were separated on a continuous sucrose gradient (20% to 50%, fraction 1; top, fraction 12; bottom). An aliquot of each fraction was analyzed by SDS-PAGE. The distributions of GRP94, BiP, Golgi p58 and calnexin were determined by Western blotting. (C) HeLa cells transiently expressing the mutant BiP with the KDEL sequence deleted were collected and homogenized. The PNS from those cells were separated on a continuous sucrose gradient (20% to 50%, fraction 1; top, fraction 12; bottom). An aliquot of each fraction was analyzed by SDS-PAGE. The distributions of the GRP94, myc-tagged mutant BiP, Golgi p58 and calnexin were determined by Western blotting. (D) Wild-type MEFs and BiP MEFs were treated with tunicamycin (2.5 ug ml^−1^ for 12, 24 h) or chloroquine (50 uM for 12 h). Cells were subjected to Western blot analysis with an anti-KDEL mouse mAb for BiP, an anti-HA mouse mAb for mutant BiP, an anti-CHOP rabbit antiserum, an anti-LC3A antiserum, and an anti-tubulin mAb.

We examined the effect of ER stress on the distribution of BiP, an endogenous ligand of the KDEL receptor, in the secretory pathway by sucrose gradient analysis in Hela cells (Figure 6A). While most BiP was found in the ER in the resting state, a significant amount could be detected in the post-ER fractions in these cells during ER stress when treated with tunicamycin (2.5 ug/ml for 24 h) which disrupts protein glycosylation in the ER, thereby inducing the UPR (Figure 6B). Then, we examined the effect of deletion of the retrieval signal from BiP. A myc-tagged mutant BiP with the KDEL sequence deleted was transiently expressed in HeLa cells. While some of the mutant BiP were localized in the ER, significant amounts were also found in the post-ER fractions (Figure 6C), suggesting that a certain fraction of BiP was transported from the ER and retrieved from the post-ER by the KDEL receptor [13]. Thus, the deletion of the retrieval signal from BiP may results in mis-sortings of cargo proteins to the secretory pathway out of the ER.

Loss of the retrieval signal may also cause a failure of the activation of the KDEL receptor. Since KDEL receptor activation has been reported to promote autophagy and removal of aggregated proteins, including SOD1 [14], we examined whether autophagy induced in the mutant BiP cells (Figure 6D). We found ER stress caused by tunicamycin treatment induced the expression of BiP in the wild type mouse embryonic cells (MEF) as well as that of the mutant BiP in the mutant MEF, while autophagy was induced only in the wild type MEF accompanying the accumulation of light chain 3AII (LC3AII).

**Figure 7:**
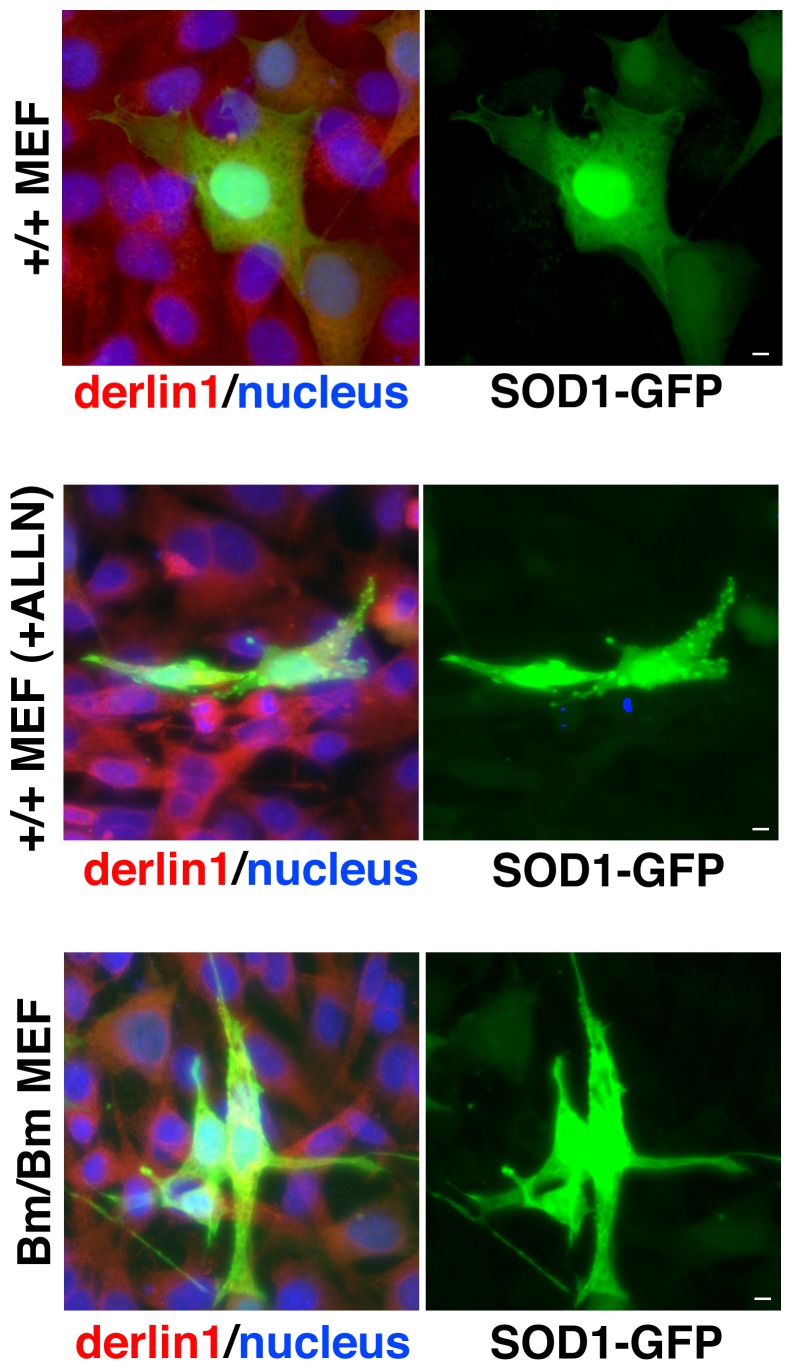
Aggregations were obvious in the mutant BiP MEF. The aggregations by transient expressions of SOD1-GFP were evaluated by immunofluorescence microscopy with labeling by using a rabbit anti-Derlin1 antibody for the ER staining (red) and SOD1-GFP (green) in wild type (+/+) and the homozygous mutant (Bm/Bm) MEF with Hoechst 33342 for nuclear staining. Scale bars, 10 um. Aggregations of SOD1were observed in the mutant BiP MEF as well as in the wild type MEF treated with a proteasome inhibitor, ALLN (10 ug/ml), at 37°C for 12 h.

If the lack of the KDEL sequence from BiP affects autophagy, cytosolic aggregates might be accumulated in the homozygous mutant BiP MEF. We over-expressed SOD1-GFP in the wild type and the homozygous mutant BiP MEF. While SOD1 distributed through the cytoplasm in the wild type MEF, it accumulated in the perinuclear space and some aggregations were found in the cytoplasm of the mutant BiP MEF (Figure 7).

Thus, lack of the KDEL sequence from BiP may lead to some functional defects of the ER chaperone in quality control and autophagy, resulting in the late onset of neurodegenerative diseases.

## Discussion

Some of the mutant BiP mice revealed motor disabilities in aging. We found a degeneration of some motoneurons in the spinal cord accompanied by the accumulation of ubiquitinated proteins. BiP, also called the 78-kD glucose-regulated protein (GRP78), is a member of the heat shock protein 70 family of proteins and is one of the most abundant ER chaperones, assisting in protein translocation, folding, and degradation [15]. Quality control in the early secretory pathway is a ubiquitous mechanism for adapting to ER stress, and the KDEL receptor and BiP are essential components of this system [13]. Proper ER-to-Golgi transport, and the subsequent retrieval of proteins and lipids to the ER, are thought to contribute to quality control [13], [16]. Deletion of the retrieval sequence from the BiP, and the consequent lack of mutant BiP recycling by the KDEL receptor, could have possible effects on protein folding in the ER and post-ER.

Mutant BiP were mostly localized to the ER, and the expression was enhanced by tunicamycin in the mutant BiP MEF [9]. A significant fraction of the mutant BiP was secreted from the cells even in the resting state, reflecting a deletion of the KDEL sequence and an impaired retrieval of mutant BiP [9]. The KDEL proteins are ER chaperones; therefore, their expression is induced extensively upon ER stress, which may cause the saturation of the KDEL receptor-mediated retrieval. A fraction of the wild-type BiP was also secreted into the medium upon ER stress caused by the tunicamycin treatment, indicating that retrieval by the KDEL receptor is saturable [9].

The homozygous mutant BiP mice have defects in some professional secretory cells. They survive only several hours after birth due to impaired pulmonary surfactant biosynthesis by alveolar type II epithelial cells causing respiratory failure [9]. They also have cortical dysplasia in the brain due to impaired synthesis of reelin by Cajal-Retzius cells. Thus, the retrieval function of BiP is essential in vivo. However, mouse embryonic fibroblasts from the homozygous mutant BiP mice are viable. Therefore, living as a whole animal may suffer from various insults from the environment.

The accumulation of misfolded proteins is one of the most common features in neurodegenerative diseases. One possible explanation for the late onset of sporadic forms of neurodegeneration is a decline, with aging, of the cellular machinery to cope with protein overload in the ER. The expression level of BiP decreases, and its chaperone function is progressively reduced due to age-related oxidization reported in rodents [10], [17]. The mutant BiP with impaired retrieval seemed to fail the induction of autophagy, which might further limit the capacity of ER quality control.

The heterozygous mutant BiP mice grew up to be adults and showed apparently normal organ development. However, vulnerability to ER stress may exist, and that vulnerability could result in chronic organ injuries with aging such as renal tubular-interstitial injury, as described in our previous work [18]. We are also interested in the relationship between ER stress and neural degeneration due to aging, such as Alzheimer's disease [19], Parkinson's disease [20], and amyotrophic lateral sclerosis [11]. The involvement of impaired BiP function in neurodegenerative diseases has been reported in a mouse model where the disruption of SIL1, a co-chaperone of BiP, causes protein accumulation and neurodegeneration [21].

Interestingly, we previously found disordered layer formation in the cerebral cortex and cerebellum in the homozygous mutant BiP neonates. Among proteins involved in corticogenesis, we found that the expression of reelin, secreted by Cajal-Retzius cells [22] was markedly reduced in the mutant brain [23]. Several studies have suggested the possible role of reelin in the pathogenesis of human mental disorders such as schizophrenia, autism, bipolar disorder, and Alzheimer's disease [24], [25]. Reelin and ApoE share ApoER2 on the cortical neurons [26], and ApoE inhibits reelin signaling by competing for binding to ApoER2. The E4 allele of ApoE increases the risk of developing sporadic forms of Alzheimer's disease. Because reelin signaling through ApoER2 in adult brains modulates synaptic plasticity and memory formation [27], a defective reelin signaling pathway may contribute to the pathogenesis of adult mental disorders. Thus, reelin signaling and ER quality control may be related to the pathogenesis of adult mental disorders, as seen in the reeler mutant–like cerebral malformation in the homozygous mutant BiP neonates [23].

The administration of chemical chaperones that promote protein folding in the ER has been reported to be effective in treating type2 diabetes, which has been shown, in experiments using a mouse model, to be connected to ER stress [28]. We have also shown that a chemical chaperone prevented the development of morphine tolerance caused by ER stress [29]. Our present study suggests that ER chaperones would be promising therapeutic targets in the treatment of chronic neurodegenerative diseases.

## Methods

### Cells and reagents

Mouse embryonic fibroblasts (MEFs) were prepared from 13.5-day-old embryos [9]. MEFs and HeLa cells were grown in a complete medium that consisted of Dulbecco's modified Eagle's medium (DMEM; Sigma Chemical Co., Irvine, UK) with 10% fetal bovine serum, 2 mM glutamine, 50 µg/ml streptomycin and 50 U/ml penicillin G at 37°C in a 5% CO_2_ incubator.

The following antibodies were used: rabbit antiserum against CHOP/GADD153, rabbit antiserum against ubiquitin, rabbit antiserum against superoxide dismutase 1 (Santa Cruz Biotechnology), rabbit antiserum against choline acetyltransferase (Millipore), rabbit antiserum against glial fibrillary acidic protein (GFAP, DakoCytomation), rabbit antiserum against Derlin-1 (MBL), mouse mAb 9E10 against the myc epitope (ATCC), mouse mAb against g-tubulin, mouse mAb against Golgi p58 (Sigma Chemical), mouse mAb SPA-827 against BiP (KDEL sequence, Stressgen), mouse mAb AF8 against calnexin (kindly provided by MM. Brenner, Boston, MA), mouse mAb 15E6 against HA tag (kindly provided by VW Hsu, Boston, MA). rabbit polyclonal antiserum against light chain (LC) 3A (Cell Signaling), Cy-2-conjugated donkey antibody against rabbit IgG, Cy-3-conjugated donkey antibody against rabbit IgG and Cy-3-conjugated donkey antibody against mouse IgG (Jackson Immunoresearch Laboratories). Tunicamycin was purchased from Nacali tasque. Hoechst 33258 was purchased from Invitrogen. Chloroquine was purchased from Sigma-Aldrich.

### Plasmids and transfection

A cDNA for the BiP deleted with the KDEL sequence was obtained by inserting a stop codon into a rat BiP cDNA (a gift from Dr. H.R.B. Pelham, MRC Laboratory of Molecular Biology, UK) just before the region encoding the carboxyl terminal KDEL sequence with a PCR reaction. The PCR product was subcloned into a pcDNA3.1 myc-His vector (Invitrogen). A PCR-generated fragment corresponding to the wild-type human SOD1 cDNA from human cDNA library was cloned into the plasmid pGFP vector (Clontech). The DNA sequences were verified using the Applied Biosystems ABI Prism 310 genetic analyzer. Transfection was performed with Fugene 6 (Roche, Basel, Switzerland).

### Mutant BiP mice

All animal experimental procedures were in accordance with a protocol approved by the Institutional Animal Care Committee of Chiba University, Chiba, Japan. A rat BiP cDNA was used as a probe to isolate a genomic clone containing the whole exon of the BiP gene from the 129/SvJ mouse genomic library in lFIXII (Stratagene). A targeting vector was used for electroporation into R1 EScells. We used homologous recombination to establish knock-in mice expressing BiP lacking the carboxyl-terminal KDEL sequence [9]. The missing KDEL sequence was replaced by a hemagglutinin (HA) tag. The resulting male chimeras were mated to C57BL/6 females. The mutant-BiP mice were investigated on the compound genetic background between the 129/SvJ derived from R1 ES cells and C57BL/6J. The heterozygotes were intercrossed to generate homozygous mutants. This hybrid line was maintained by brother-sister mating for at least ten generations.

### Western blotting

Cultured cells were washed twice with ice-cold PBS and then homogenized in a buffer containing 0.4% (w/v) Nonidet P-40, 0.2% N-lauroylsarcosine, 30 mM Tris/HCl pH 8.0, 1 mM EDTA, 10 µg/ml aprotinin, 10 µg/ml leupeptin, 30 µg/ml N-acetyl-l-leucinal-l-lecinal-l-norleucinal (ALLN, Sigma Chemical). The mice were deeply anesthetized with pentobarbital (Dainippon Sumitomo Pharma). Brains and spinal cords were homogenized by sonication (UR-20P, TOMY, Tokyo, Japan) in a buffer containing 0.4% (w/v) Nonidet P-40, 0.2% N-lauroylsarcosine, 30 mM Tris/HCl pH 8.0, 1 mM EDTA, 10 µg/ml aprotinin, 10 µg/ml leupeptin, 30 µg/ml ALLN.

The lysates were centrifuged, and the supernatants were resuspended in SDS-PAGE sample buffer and then separated by SDS-PAGE under reducing conditions. The proteins were transferred from the gels to polyvinylidene fluoride membranes (Immobilon-P, Millipore Corp.), and Western blotting was done as previously described [30]. Imaging was obtained by LAS-1000 and Image Gauge software (Fuji Photo Film Co. Ltd.).

### Sucrose gradient experiment

A postnuclear supernatant was obtained and loaded on a continuous sucrose gradient (20–50%), as described previously [13]. Twelve fractions were obtained from each sample. An aliquot of each fraction was separated by SDS-PAGE under reducing conditions, and the distribution of each protein was determined by Western blotting, developed by chemiluminescence (ECL, Amersham Pharmacia Biotech). Imaging was obtained by LAS1000 and Image Gauge software (Fuji Photo Film Co. Ltd., Tokyo, Japan).

### Immunohistochemistry

The mice were deeply anesthetized with pentobarbital and fixed by transcardiac perfusion with 4% paraformaldehyde in phosphate-buffered saline (PBS). The spinal cords were further immersion-fixed for 12 h in 4% paraformaldehyde at 4°C. After fixation, they were dehydrated in increasing concentrations of ethanol and embedded in paraffin wax. For the immunofluorescence, the sections (8 µm) were incubated with 10% normal goat or bovine serum in PBS for 30 min to block nonspecific antibody binding, and then incubated with a primary antibody in PBS for 1 h at room temperature. The sections were rinsed with PBS and then incubated with a mixture of Cy3-conjugated anti-rabbit IgG and Cy2-conjugated anti-mouse IgG in PBS for 1 h at room temperature. Then, the sections were rinsed with PBS and mounted on glass slides with Perma Fluor (Immunon). Immunolocalization was observed with a fluorescence microscope using FITC/rhodamine filters and a Plan-Neofluar 20× and 40× NA 0.75 objective (Axiovert 200 M, Carl Zeiss). The brightness and contrast were optimized by AxioVision 4.4 software (Carl Zeiss), and the immunofluorescence images were captured with a digital camera (AxioCam MRm, Carl Zeiss). For the immunohistochemistry, the spinal cord sections were incubated with 10% normal goat or bovine serum in PBS for 30 min to block nonspecific antibody binding, and then incubated with a primary antibody in PBS for 12 h at 4°C. The sections were rinsed with PBS, incubated with a secondary antibody in PBS for 2 h at room temperature, and then visualized using the VECTASTAIN Elite ABC kit (Vector Laboratories) with diaminobenzidine (Sigma).

### TUNEL staining

The mice were deeply anesthetized with pentobarbital (Dainippon Sumitomo Pharma). The spinal cords were isolated, and apoptotic cells were visualized by TUNEL assay based on the manufacturer's protocol (Roche) as previously described [30]. The TUNEL staining was observed with a microscope using a N-Achroplan 40× NA 0.65 objective (Axio Imager A1, Carl Zeiss). The brightness and contrast were optimized by AxioVision Rel.4.7 software (Carl Zeiss), and the images were captured with a digital camera (AxioCam MRc, Carl Zeiss).

### Statistical analysis

To compare values between groups, Chi-square and Fisher's exact test (Figure. 1) and t-test (Figure 3B) were used (GraphPad Prism 4.0, GraphPad Software, San Diego, CA). Statistical significance was accepted at *P*<0.05.

## Supporting Information

Video S1 **A seventeen month-old heterozygous mutant BiP mouse displayed paralysis.**
(MP4)
